# Chloroform and Its Administration

**Published:** 1866-09

**Authors:** 

**Affiliations:** Burlington, Iowa


DENTAL REGISTER.

VOL. XX.] SEPTEMBER, 1866. [No. 9.

### Original Communications.

### CHLOROFORM AND ITS ADMINISTRATION.

BY DR. COCHRAN, OF BURLINGTON, IOWA.

Read before the Iowa State Dental Society, July, 1866.

THIS substance was discovered by Mr. Samuel Guthrie, of Sackett's Harbor, New York, in 1831, and about the same time by Soubeiran, in France, and Liebig, in Germany.

Guthrie obtained it by distilling a gallon from a mixture of three pounds of chloride of lime and two gallons of alcohol, and rectifying the product by redistillation, first from a great excess of chloride of lime, and afterwards from carbonate of potassa. It is thus evident that Guthrie obtained in a pure state the substance now called chloroform; but he erroneously supposed his product to be the well known oily liquid of the Dutch chemist, which it greatly resembles. Mr. Guthrie called the substance, in his communications, chloric ether, one of the names by which the Dutch liquid, or chloride of alifiant gas, is designated. He was induced to make the preparation from noticing a passage in Professor Silliman's Elements of Chemistry, which referred to the Dutch liquid as a grateful diffusible stimulant, when properly diluted with alcohol and water. In relation to the anticipated importance of this substance as a medicine (not as an anæsthetic agent, at that early day, for its legitimate use was not then at all understood), Mr. Daniel B. Smith, President of the Philadelphia College of Pharmacy, holds the following language, in July, 1832:

"The action of this ether on the living system is interesting, and may hereafter render it an object of importance in commerce. Its flavor is delicious, and its intoxicating qualities equal to or surpassing those of alcohol. It is a strong diffusible stimulant, similar to the hydrated ether, but more grateful to the taste."

ITS ADMINISTRATION.

It should never be given but by a person accustomed to its use, and well acquainted with its effects upon the living system. The following is the way, I think, chloroform may be most conveniently and safely administered:

Take a small linen handkerchief and pack it into a rounded ball, with convex and concave surfaces. Hold the convex in the palm of the hand, and pour about ʒi. of chloroform in the center of the concave part of the ball, which will be absorbed by the handkerchief immediately, and given off by evaporation slowly or more rapidly, just as you close or open the hand. The patient should be admonished not to eat anything of a gross character previous to the use of chloroform, say six or eight hours, nor but very little of anything for three or four hours before its use. Place the patient on the chair in a recumbent position, or as near so as possible; hold your hand containing the handkerchief saturated with the chloroform about one inch from the patient's nose and mouth, being careful to let there be a free admixture of atmospheric air. At first it will seem to be oppressive, but the patient will soon become accustomed to its use, and make her or his inspirations free and easy.

The principal points to be attended to during inhalation of this potent agent are, that it should not be given too suddenly or in too concentrated a form, and while under its influence the patient be not raised up in the chair. Care should be taken not to compress the abdomen in holding the patient, for as respiration becomes chiefly or wholly diaphragmatic, it may be seriously interrupted by any pressure on the abdominal walls. Whilst under its influence the patient should never be raised up, as has just been stated, for as this agent exercises a powerful sedative action on the heart, sudden and perhaps fatal syncope may ensue by putting the patient in the erect position.

With due caution it may be given with perfect safety to individuals of all ages. In administering it to young children, Dr. Snow recommends that it should be diluted with rectified spirits. The first influence of chloroform appears to be exercised upon the nervous system. The patient becomes excited and talkative, and a state of unconsciousness is induced, the muscular system at the same time being rendered rigid and tense. At this time the heart's action is usually quickened, and more forcible than natural.

As the administration of chloroform continues, however, complete paralysis of sense and motion is induced. The patient becomes altogether unconscious to all external impressions, the muscles becoming relaxed and the action of the heart slow and feeble.

It should be borne in mind that it is not necessary in all operations to administer chloroform; but that there are cases that justify its use must be patent to every one. Some good dentist doubtless will oppose the use of chloroform because *accidents* have occurred from its reckless and indiscriminate use, others from a sense of *fear* and prejudice in favor of some other anæsthetic agent. Why should there be such a hue and cry raised against this invaluable agent? Show me a drug in the Materia Medica that possesses potent and reliable properties, and I will show you a drug that has killed more patients than ever was dreamed of having *died* from the use of chloroform. Yes, gentlemen, there is no valuable medicine known in the catalogue of drugs, if improperly applied, but what will have a deleterious and baneful effect. Take, for instance, all the preparations of opium, quinine, digitalis, veratrum viride, strychnia, lobelia, antimony, calomel, and hundreds of other articles in daily use. They, it is true, have ameliorated the condition of thousands, yet at the same time they have slain their hundreds. Yet none think of discarding them, or ruling them out of use when indication calls for their administration.

